# Characteristics of Doctors and Others

**Published:** 1854-11

**Authors:** 


### CHARACTERISTICS OF DOCTORS AND OTHERS.

Tasso's conversation was neither gay nor brilliant.

Dante was either taciturn or satirical.

Butler was sullen or biting.

Gray seldom talked or smiled.

Hogarth and Swift were very absent-minded in company.

Milton was unsociable and even irritable when pressed into conversation.

Kirwan, though copious and eloquent in public addresses, was meagre and dull in colloquial discourse.

Virgil was heavy in conversation.

La Fontaine appeared heavy, coarse, and stupid; he could not speak and describe what he had just seen, but then he was the model of poetry.

Chaucer's silence was more agreeable than his conversation.

Dryden's conversation was slow and dull, his humor saturnine and reserved.

Descartes was silent in mixed company.

Corneille, in conversation was so insipid that he never failed in wearying. He did not even speak correctly that language of which he was such a master.

Ben Johnson used to sit silent in company and suck *his* wine and *their* humors.

Southey was stiff, sedate, and wrapped up in asceticism.

Addison was good company with his intimate friends, but in mixed company he preserved his dignity by a stiff and reserved silence.

Junius was so modest that he could scarcely speak upon the most common subjects without a suffusion of blushes.

Fox, in conversation never flagged; his animation and variety were inexhaustible.

Dr. Bentley was loquacious.

Grotius was talkative.

Goldsmith wrote like an angel, and talked like a poor Poll.

Burke was eminently entertaining, enthusiastic and interesting in conversation.

Curran was a convivial deity; he soared into every region, and was at home in all.

Dr. Birch dreaded a pen as he did a torpedo; but he could talk like running water.

Dr. Johnson wrote monotonously and ponderously, but in conversation his words were close and sinewy; and if his pistol missed fire, he knocked down his antagonist with the butt of it.

Coleridge, in his conversation, was full of acuteness and originality.

Leigh Hunt has been well termed the philosopher of Hope, and likened to a pleasant stream in conversation.

Carlyle doubts, objects, and constantly demurs.

Fisher Ames was a powerful and effective orator, and not the less distinguished in the social circle. He possessed a fluent language, a vivid fancy, and a well stored memory.

